# A functional variant in GREM1 confers risk for colorectal cancer by disrupting a hsa-miR-185-3p binding site

**DOI:** 10.18632/oncotarget.18095

**Published:** 2017-05-23

**Authors:** Jiaoyuan Li, Hui Liu, Li Zou, Juntao Ke, Yi Zhang, Ying Zhu, Yang Yang, Yajie Gong, Jianbo Tian, Danyi Zou, Xiating Peng, Jing Gong, Rong Zhong, Kun Huang, Jiang Chang, Xiaoping Miao

**Affiliations:** ^1^ Department of Epidemiology and Biostatistics, School of Public Health, Tongji Medical College, Huazhong University of Science and Technology, Wuhan, China; ^2^ National Key Laboratory of Crop Genetic Improvement, Huazhong Agricultural University, Wuhan, China; ^3^ Department of Health Care, Bao’an Maternal and Child Health Hospital, Shenzhen, China; ^4^ Tongji School of Pharmacy, Huazhong University of Science and Technology, Wuhan, China; ^5^ Key Laboratory of Environment and Health, Ministry of Education & Ministry of Environmental Protection, Tongji Medical College, Huazhong University of Science and Technology, Wuhan, China

**Keywords:** TGF-β, GREM1, untranslated region, microRNA, colorectal cancer

## Abstract

The transforming growth factor beta (TGF-β) pathway has been implicated in carcinogenesis of intestinal canal. Except for common variants indentified by genome-wide association studies, variants with lower frequency can also explain a part of the disease heritability, especially those in gene regulatory regions. In this study, we searched for colorectal cancer (CRC) related functional low-frequency variants (minor allele frequency 1-5%) in untranslated regions (UTR) involved in the TGF-β signaling using a next-generation sequencing based approach. A case-control study including 1,841 CRC cases and 1,837 controls was performed to identify CRC associated variants and biological experiments were applied to further explore the potential functions of the significant variants. Three low-frequency UTR variants were selected as our candidates and subsequent association analyses showed that a low-frequency variant rs12915554 in the 3’ UTR of GREM1 was significantly associated with CRC risk (Additive model: OR=1.43, 95%CI: 1.04-1.95, *P*=0.026). Functional annotations suggested that rs12915554 variation increased the expression of GREM1 by perturbing a hsa-miR-185-3p binding site. Moreover, higher expression level of GREM1 was investigated in colon tumor tissues compared with adjacent normal tissues using TCGA data. In conclusion, low-frequency UTR variant rs12915554 in the gene GREM1 was in relation to CRC susceptibility in a Chinese population and this variation might promote CRC development through enhancing GREM1 expression in a miRNA-mediated posttranscriptional manner.

## INTRODUCTION

Colorectal cancer (CRC) is one of the most commonly diagnosed cancers worldwide, with an estimated 1.4 million cases and 693,900 deaths occurring in 2012 [1]. In China, the incidence of CRC had a significant upward trend in both males and females owing to westernized lifestyle and aging of the population [2], highlighting the urgency in CRC prevention. In addition to environmental factors like smoking, diet and obesity [3–5], inherited genetic factors has also been well established in CRC etiology [6, 7].

Previous association studies based on candidate approach and large scale genome-wide association studies (GWAS) have identified a battery of genetic variants with propensity to CRC development [8–11]. In particular, GWAS have renewed the field of genetics for complex diseases and have contributed to unprecedented advances in our understanding of the role of common variation in CRC. To date, this comprehensive and powerful approach has demonstrated more than 50 genetic loci which accounting for CRC susceptibility in different populations [12]. Among these, six newly identified loci were located in or were near to genes of the transforming growth factor beta (TGF-β) superfamily, including TGFB1, SMAD7, BMP2, BMP4, CDH1 and GREM1 [13–19]. The perturbation of the TGF-β signaling pathway suggested an important role of this pathway in CRC generation and progression. However, the heritability explained by over 20 known common CRC associated single nucleotide polymorphisms (SNP) identified by GWAS was less than 1%. Meanwhile, the heritability explained by all common SNPs was similarly as low as 7.4%, suggesting that much of the genetic architecture to CRC remains to be detected [20].

Although common variants are the major contributors to CRC susceptibility, a fraction of the disease heritability may be explained by other genetic variants with lower minor allele frequency (MAF), that is, low frequency variants (MAF: 1-5%) and rare variants (MAF<1%). These variants which ignored by GWAS may be also a source of functional variants which accounting for disease risk. To test this hypothesis, we have comprehensively evaluated the genetic effects of low frequency variants involved in the TGF-β signaling pathway on CRC susceptibility using a next generation sequencing based approach. In our previous study, we focused on nonsynonymous coding variants and found that a low-frequency missense variant rs3764482 in SMAD7 was significantly associated with CRC risk in a Chinese population [21]. However, eukaryotic untranslated region is critical for recruitment of transcription factors and noncoding RNAs, thus plays a major role in regulating gene transcription and translation [22, 23]. In this study, we expanded our attention to the role of low-frequency UTR variants located in key genes of the TGF-β pathway in CRC development. Among the 17 UTR variants initially captured by our targeted sequencing, 3 were defined as low-frequency variants based on their frequencies in our subjects. Thus we selected these 3 eligible variants as our candidates and conducted a case-control study to identify genetic variants in relation to CRC susceptibility in a Chinese population. Besides, biological experiments were introduced to further explore the potential functions of the significant variants and uncover the underlining pathogenic mechanisms.

## RESULTS

### Associations between candidate variants and CRC susceptibility

The basic characteristics of the subjects enrolled in this case-control study were reported previously [21]. Briefly, no significant differences were found between cases and controls in distribution of gender, age and drinking status. However, smoking was significantly associated with an increased risk of CRC (data not shown).

The three candidate SNPs included in this study were located in the 3’ UTR of the gene gremlin 1 (GREM1) and the basic information about these SNPs were shown in Supplementary Table 1. One of the candidate SNPs, rs117841568, was failed in design of the genotyping probes. Consequently, we examined associations between the remaining two candidate SNPs, rs12915554 and rs146588909, and CRC risk in our subsequent study. The two SNPs included in our study had an average genotyping call rate of 97.9% and none of them deviated from Hardy-Weinberg equilibrium (HWE). There was no significant association between rs146588909 and risk of CRC (OR=0.80, 95%CI: 0.37-1.62, *P*=0.571 under a dominant model). As for the other SNP, we found that the minor allele of rs12915554 was significantly associated with increased CRC risk. Compared with the wild allele C, the OR for the minor allele A was 1.44 (95%CI: 1.05-1.98, *P*=0.024). Moreover, the association between this variant and CRC risk was significant both in additive model (OR=1.43, 95%CI: 1.04-1.95, *P*=0.026) and dominant model (OR=1.43, 95%CI: 1.04-1.97, *P*=0.029). All the detailed results were presented in Table 1.

**Table 1:** Associations between candidate SNPs involved in TGF-β signaling and CRC risk

Gene	rs	Genotype	Cases (%)	Controls (%)	OR*	95%CI	*P*	*P*_hwe_
GREM1	rs12915554	CC	95.0	96.3	1.00			1.000
		CA	4.8	3.7	1.37	0.99-1.89	0.058	
		AA	0.2	0.0	˗			
		CA+AA	5.0	3.7	1.43	1.04-1.97	0.029	
		Additive model			1.43	1.04-1.95	0.026	
		Allelic model			1.44	1.05-1.98	0.024	
GREM1	rs146588909	AA	99.3	99.2	1			1.000
		AG	0.7	0.8	0.80	0.37-1.62	0.571	
		GG	0.0	0.0	˗			
		AG+GG	0.7	0.8	0.80	0.37-1.62	0.571	
		Additive model			0.80	0.37-1.62	0.571	
		Allelic model			0.80	0.38-1.72	0.572	

*: Adjusted for age, sex, smoking and drinking status.

### The SNP rs12915554 enhanced GREM1 mRNA expression

**Figure 1:**
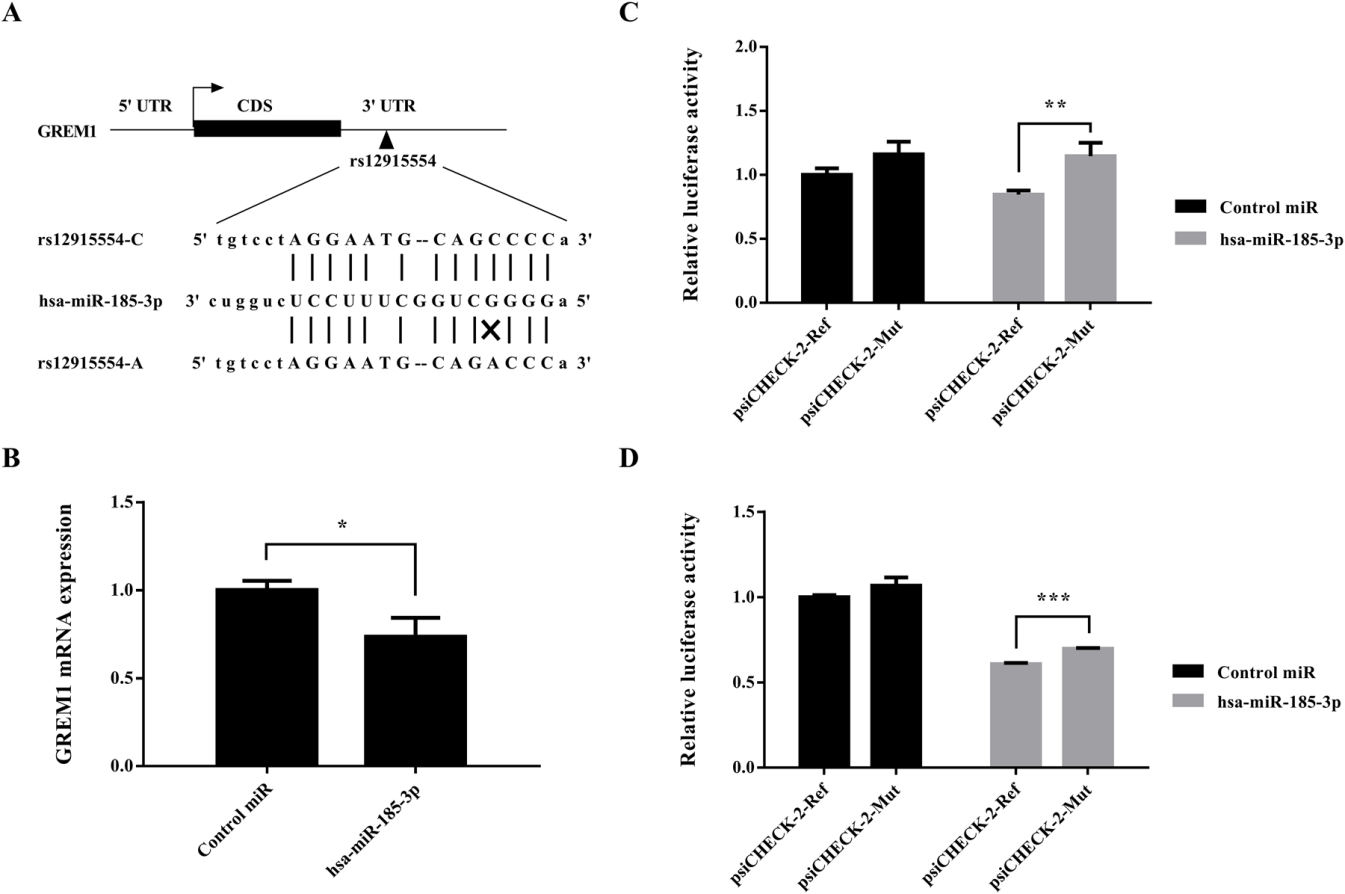
GREM1 rs12915554 enhanced GREM1 expression through disrupting a hsa-miR-185-3p binding site **(A)** *In silico* prediction of the hsa-miR-185-3p binding site in GREM1 mRNA. Putative rs12915554 function was determined using the miRNASNP 2.0. **(B)** GREM1expression was suppressed by the stimulation of hsa-miR-185-3p. **(C)** The relative luciferase activity was enhanced in rs12915554 risk A allele comparing with the reference C allele in LoVo cells, with the treatment of hsa-miR-185-3p. **(D)** The relative luciferase activity was enhanced in rs12915554 risk A allele comparing with the reference C allele in SW480 cells, with the treatment of hsa-miR-185-3p. Data are represented as mean±SD and the error bar represents the standard deviation. *: *P*<0.05, **: *P*<0.01, ***: *P*<0.005.

*In silico* analyses showed that the C>A change of SNP rs12915554 disrupted a putative binding site for hsa-miR-185-3p (Figure 1A). Therefore, rs12915554 might affect CRC susceptibility via controlling GREM1 gene expression. Since microRNAs often function as negative regulators of gene expression, we first investigated whether the GREM1expression was influenced by the stimulation of the promising microRNA hsa-miR-185-3p. As expected, the treatment of microRNA hsa-miR-185-3p on colon cancer cells reduced the expression of GREM1 when compared with the treatment of negative control (*P*=0.019, Figure 1B). This suggested a negative regulation of microRNA hsa-miR-185-3p on GREM1 mRNA expression level.

Accordingly, we hypothesized that the rs12915554 variation might accelerate GREM1 expression in a microRNA-mediated posttranscriptional manner. To validate this hypothesis, we next performed luciferase assays to determine the effect of rs12915554 on GREM1 expression. The discrepancy of relative luciferase activity between wild allele group and minor allele group was non-significant when the cells were treated with control microRNA. Under the treatment of hsa-miR-185-3p, the relative luciferase activity was significantly increased in rs12915554 risk allele group comparing with the reference group in both LoVo cells and SW480 cells (*P*=0.009 for LoVo cells and *P*=6.8×10^-5^ for SW480 cells, Figure 1C and 1D). This was consistent with the result that over-expression of hsa-miR-185-3p caused a reduction of endogenous GREM1 mRNA level which we just mentioned above.

### Over-expression of GREM1 in colon cancer tissues

**Figure 2:**
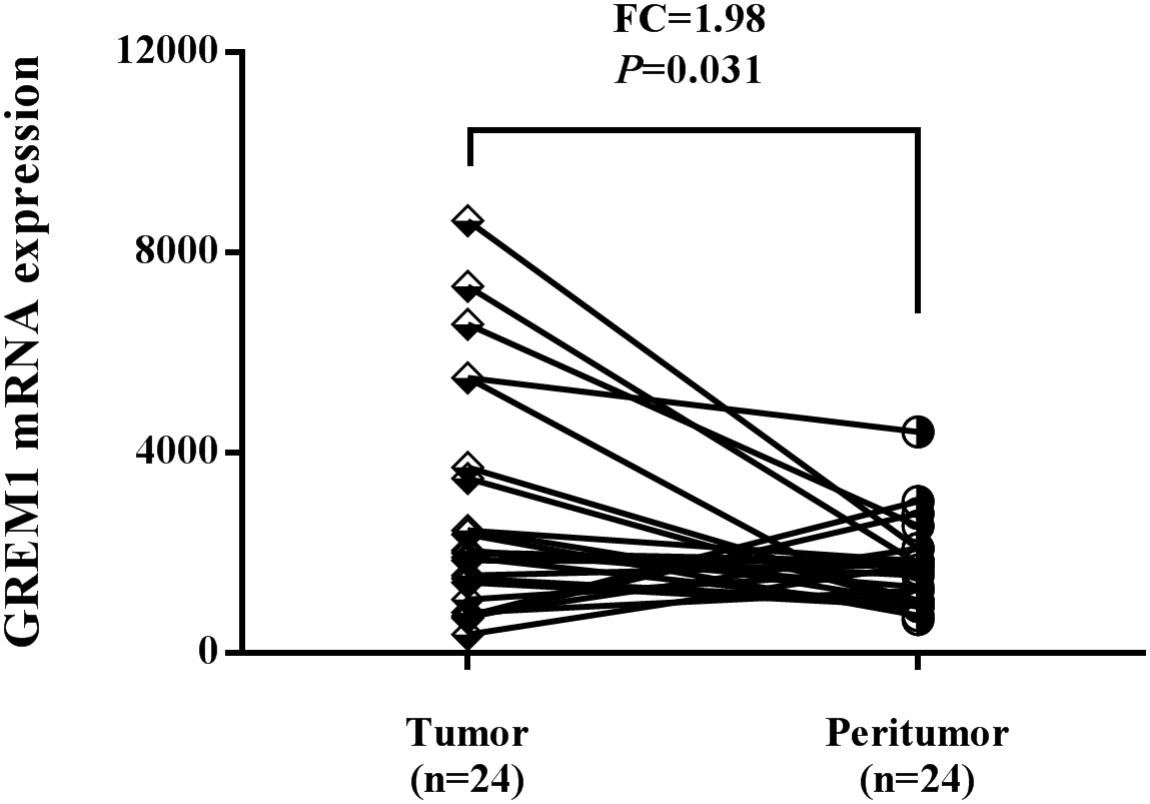
The expression level of GREM1 was increased in colon tumor tissues compared with adjacent normal tissues using TCGA dataset

Considering the conspicuous genetic effects of rs12915554 variation on CRC susceptibility and its positive role in controlling GREM1 expression, we next evaluated the GREM1 expression in colon tumor tissues and adjacent normal tissues using data downloaded from The Cancer Genome Atlas (TCGA) database. Higher GREM1 mRNA expression was observed in colon tumor tissues compared with adjacent normal tissues. The fold change of tumor to normal tissues reached as high as 1.98 (Figure 2, *P*=0.031).

## DISCUSSION

A major challenge of post-GWAS era is to explain the “missing heritability” and further identify novel functional variants accounting for complex diseases. With a next-generation sequencing based approach, we previously screened out a CRC associated low-frequency coding variant which involved in the TGF-β signaling pathway [21]. In the current study, we focused on the role of UTR variants located in the same pathway and found a low-frequency UTR variant GREM1 rs12915554 was significantly associated with CRC risk in a Chinese population. Functional analyses showed that the C>A change of rs12915554 disrupted a binding site for hsa-miR-185-3p and enhanced GREM1 mRNA expression by blocking microRNA mediated degradation. In addition, the expression of GREM1 was elevated in CRC tumors. Taken together, our study suggested that low-frequency variant rs12915554 within GREM1 contributed to CRC susceptibility and it might promote CRC development by enhancing GREM1 gene expression.

Low-frequency and rare variants ignored by GWAS and imputation might also confer functional effects in some disease patients [24]. Owing to low risk allele frequencies, these cancer predisposing loci were difficult to identify and their detection required re-sequencing of the entire associated regions [25]. Using an approach combining sequencing of the informed candidate genes and a case-control study with large sample size, we identified a low-frequency UTR variant GREM1 rs12915554 newly linked to CRC risk in a Chinese population. GWAS have identified several CRC associated SNPs in the SCG5-GREM1 region mapped to chromosome 15q13.3 [12]. For example, an intergenic variant near GREM1, rs4779584, was one of the earliest variants to be found in responsible for CRC risk [26]. Among those CRC SNPs identified by previous GWAS, no SNP was in linkage disequilibrium (LD) with the significant SNP rs12915554 characterized in our study, though they were physically close to each other within the same locus. Together with the compelling functional effects of rs12915554 revealed in the current study, we proposed that the low-frequency variant rs12915554 located in GREM1 3’ UTR might be an independent novel risk variant contributing to CRC development. Besides, the MAF of rs12915554 is distinct across different populations. This variation is common in Americans (MAF: 32%) and Europeans (MAF: 36%), while in East Asians, it is a low-frequency variation with a MAF of 3%. Considering the difference of the allele frequency among diverse populations, the genetic association between rs12915554 and CRC risk might be specific in Chinese population. However, replications of our findings are warranted in the future.

As a member of the TGF-β super-family, GREM1 involves in multiple cell processes including cell growth, differentiation and development, through antagonizing the activity of bone morphogenetic proteins (BMPs). The binding of GREM1 to selective BMPs (BMP2, BMP4 and BMP7) prevents ligand-receptor interaction and blocks subsequent downstream signaling [27, 28]. It has been well established that the BMP signaling plays an important role in maintaining intestinal homeostasis and the disrupted BMP signaling can lead to intestinal polyps and sporadic CRC [29, 30]. In addition to inhibiting the canonical pathway signal through BMP receptors, GREM1 could also activate the major proangiogenic receptor VEGFR2 and thus acted as an agonist of angiogenesis [31, 32]. Aberrant epithelial expression of GREM1 has been documented in diverse carcinomas [33, 34]. Furthermore, over-expression of GREM1 initiated tumorigenesis and promoted tumor cell proliferation [33, 35]. The tumor-promoting effect of GREM1 has been highlighted by its causative role in triggering hereditary mixed polyposis syndrome (HMPS) and CRC. Jaeger et al. found that a 40-kb duplication upstream GREM1 caused a Mendelian-dominant predisposition to CRC and the underlying disease mechanism was strong, ectopic expression of GREM1 in colorectal epithelium [36]. Coincidentally, a high penetrant duplication in the regulatory region of GREM1 was also proved to be responsible for CRC susceptibility [37]. Fine-mapping of the CRC associated variants in SCG5-GREM1 locus showed that a functional SNP acted as an allele-specific GREM1 enhancer and influenced cancer risk through differential CDX2 and TCF7L2 binding [38]. Interestingly, the increased GREM1 expression was predicted to reduce the activity of BMP pathway, a mechanism which underlies tumorigenesis in juvenile polyposis [39]. These evidences suggested activation of GREM1 as a cause of initiation and development of CRC. In the present study, our functional analyses revealed that the risk allele of rs12915554 enhanced GREM1 expression by interfering a hsa-miR-185-3p binding site. These results gave further support to the oncogenic role of GREM1 in CRC development.

MicroRNAs modulate extensive biological processes mostly via posttranscriptional regulation of target genes. Usually, they silence eukaryotic gene expression through promoting the degradation or inhibiting the translation of target mRNAs [40, 41]. Alterations of microRNA-mediated regulation were implicated in essential processes of carcinogenesis, such as proliferation, angiogenesis, apoptosis and invasion [42, 43]. Whether a microRNA acts as a tumor promoter or a tumor suppressor is dependent on the presence of its targets and the cellular context. MiR-185 was found to be a tumor suppressor with repressing effects against growth and invasion of colon cancer cells through inhibiting HIF-2α signaling [44]. As a microRNA processed from the 3’ stem-loop arm of miR-185, miR-185-3p has been proposed as an inhibitor of c-Myc levels via an autoregulatory feedback mechanism [45]. This microRNA was also involved in the restraint of cancer cell growth and apoptosis by targeting SMAD7 and WNT2B, and predicted radiosensitivity of nasopharyngeal carcinoma [46, 47]. Our findings here demonstrated that the rs12915554 A allele disrupted a hsa-miR-185-3p binding site within the 3’ UTR of GREM1, and consequently increased GREM1 expression. Considering the biological effects of GREM1 and BMP signaling, we speculated that the elevated GREM1 expression drove CRC development via reducing the activity of BMP receptors and inhibiting downstream TGF-β signaling. However, the detailed functional consequences of the rs12915554 variation and the downstream effects of inactivated BMP signaling await further investigation.

To our knowledge, the causal association between GREM1 rs12915554 and CRC risk was first identified in this study. Our study highlights the power of low-frequency variants in explaining the missing heritability and may advance our understanding in the pathogenesis of complex diseases. Nevertheless, certain limitations should be acknowledged. First, only three candidate SNPs were included in this association study. Comprehensive approaches are required to systematically elaborate the role of low-frequency and rare variants in CRC development. Second, insufficient data about demographic and environmental factors restricted us to investigate the influences of other risk factors like diet, obesity and physical activity in our analyses. More environmental risk factors should be considered and replications of our study are needed in the future. Third, although we have explored the primary function of rs12915554 on GREM1, the detailed biological consequences of this variation and downstream effects still remain unclear. Further studies of the underlying mechanisms are warranted.

In conclusion, low-frequency 3’ UTR variant rs12915554 in GREM1 contributed to CRC susceptibility in a Chinese population. Functional annotations provide limited but suggestive evidence that rs12915554 variation ascended the expression of GREM1 through perturbing a hsa-miR-185-3p binding site. The genetic association and function exploration in our study support the suggested role of activated GREM1 as a cause of initiation and development of CRC.

## MATERIALS AND METHODS

### Study subjects

A total of 1,841 CRC cases and 1,837 healthy controls were included in this case-control study. The CRC cases were recruited between March 2009 and March 2011 from two hospitals: the Tongji Hospital of Huazhong University of Science and Technology in Wuhan, China and the Cancer Institute and Hospital in Beijing, China. Controls were randomly selected from healthy volunteers who visited the health check-up center in the same hospital during the same period that the CRC patients were recruited. The selection criteria for CRC cases and controls were described before and all the subjects in this study were included in the stage 1 case-control study in our previous research [21].

### Selection of candidate SNPs and genotyping

The procedures for targeted sequencing and basic information about captured variants were described before [21]. Briefly, we have sequenced the coding exons of 12 key genes (TGFB1, TGFBR1, TGFBR2, BMP2, BMP4, BMPR1A, BMPR1B, BMPR2, SMAD4, SMAD7, CDH1, GREM1) located in the TGF-β signaling pathway, using genomic DNA isolated from blood samples of 96 CRC patients. The primary sequencing data were generated by Ion Torrent Personal Genome Machine® (PGM®) platform (Life Technologies, Carlsbad, California, USA) following the manufacturer’s protocol. All variants were visualized by the Integrative Genomics Viewer (IGV) and potential novel variants were validated with Sanger sequencing in both directions to obsolete false positive. As a result, the Ion Torrent semiconductor sequencing initially captured 78 SNPs, including 23 intron variants, 16 synonymous variants, 22 missense variants and 17 exonic UTR variants. After excluding 11 common (MAF>5%) and 3 rare (MAF<1%) UTR variants, 3 UTR variants with low frequency (MAF: 1%-5%) captured in our targeted sequencing were selected as candidates in this case-control study. Genomic DNAs were extracted from peripheral blood samples by using the RelaxGene Blood DNA System DP319-02 (TIANGEN, Beijing, China). Candidate variants were then genotyped using ABI OpenArray assays (Applied Biosystems, Foster City, California, USA) under standard conditions.

### Function prediction of the significant SNPs

The prediction of the putative functions for significant SNPs were achieved through appropriate bioinformatics resources. Specifically, we searched for the putative binding sites for rs12915554 using ANNOVAR (http://www.openbioinformatics.org/annovar/) and miRNASNP 2.0 (http://bioinfo.life.hust.edu.cn/miRNASNP2/index.php) datasets.

### Differential gene expression analysis

To investigate the difference of GREM1 gene expression levels between colon tumor and normal tissues, GREM1 mRNA expression data of 24 paired colon tumor tissue samples and adjacent normal tissue samples were downloaded from TCGA dataset (https://cancergenome.nih.gov/). And the standardized expressions of GREM1 were then compared between normal colon and tumor tissues.

### Plasmid constructions

The wild-type transcript sequence flanking GREM1 (NM_001191322) rs12915554 (±100 bp) was downloaded from the NCBI database and synthesized by Genewiz Company (Suzhou, China), then cloned into the *Xho* I and *Not* I sites of psiCHECK™-2 expression vector. The mutation transcript sequence corresponding to genetic variant rs12915554 (C>A) was generated by site-specific mutagenesis and cloned along the same strategy used for the wide type sequence. Recombinant expression vectors containing wild type (psiCHECK-2-ref) and mutant type of rs12915554 (psiCHECK-2-mut) were validated by Sanger sequencing.

### Cell culture and transfection

The human colon cancer cell lines SW480 and LoVo were obtained from the China Center for Type Culture Collection (Wuhan, China). Both of the SW480 and LoVo cell lines have never been passaged longer than 3 months. They all have been tested routinely by DNA sequencing using the Applied Biosystems AmpF/STR identifier kit and last checked in September 2016. Cells were cultivated in Dulbecco’s Modified Eagle’s Medium (DMEM, Gibco, USA) containing 10% fetal bovine serum (FBS, Gibco, USA) and 100 U/ml Penicillin-Streptomycin solution. All cells were maintained at 37°C in a humidified atmosphere with 5% CO2. Transient transfections were performed by using Attractene (QIAGEN, Germany) according to the manufacturer’s instructions.

### Quantitative real-time PCR (qRT-PCR) analysis

LoVo cells were incubated with microRNA mimics (hsa-miR-185-3p) or microRNA negative control (Control miRNA) and harvested 24h after miRNA treatment. Total RNA was extracted from the cells using TRIzol Regent (Bio Basic Inc., Canada) according to the manufacturer’s instruction. Reverse transcription was performed using MMLV RTase cDNA Synthesis Kit (TaKaRa, Japan). The level of GREM1 mRNA was measured by qRT-PCR using Power SYBR Green PCR master mixture (Applied Biosystems, USA), with GAPDH expression as the endogenous control. And the fold change was calculated by relative quantification (2^−ΔΔCt^).

### Luciferase reporter gene assay

Luciferase assay was determined following the protocol we described before. In brief, cells were seeded at 1×10^5^ cells per well onto a 24-well tissue cultured plate and incubated overnight, and then transiently transfected with luciferase vector psiCHECK-2-ref or psiCHECK-2-mut and microRNA mimics (hsa-miR-185-3p) or microRNA negative control (Control miRNA). Reporter assays were performed at 24h post-transfection using the dual-luciferase assay system (Promega, USA). Renilla luciferase and firefly luciferase activities were detected and the relative luciferase activity was calculated to compare the discrepancy between two different groups. All experiments were independently performed in triplicate.

### Statistical analysis

The HWE for each candidate variant was assessed by a goodness-of-fit χ^2^ test in the control group. The associations between variants and susceptibility of CRC were demonstrated by logistic regression (LR) with adjustment for gender, age, smoking and drinking status. Odds ratios (OR) and 95% confidence intervals (CI) were assumed in different genetic models. The Cochran-Armitage trend test was applied to evaluate the trend of relationship. We compared the mRNA expression levels of GREM1 between tumor and peritumor tissues using paired Student’s t test. For *in vitro* functional analyses, all experiments data are presented as the mean±SD and analyzed using a Student’s t test.

All the statistical analyses were conducted by SPSS Statistics and *P*<0.05 was defined as statistically significant.

## SUPPLEMENTARY MATERIALS TABLE



## Abbreviations

CRC: colorectal cancer
GWAS: genome-wide association study
TGF-β: transforming growth factor β
UTR: untranslated region
GREM1: gremlin 1
BMP: bone morphogenic protein
MAF: minor allele frequency
HWE: Hardy-Weinberg equilibrium
LR: logistic regression
SNP: single nucleotide polymorphism
HMPS: hereditary mixed polyposis syndrome
CI: confidence interval
TCGA: The Cancer Genome Atlas
