# Supplementary material for: A functional variant in GREM1 confers risk for colorectal cancer by disrupting a hsa-miR-185-3p binding site
Source: Oncotarget. 2017 May 23;8(37):61318–26. doi: 10.18632/oncotarget.18095 (PMC5617425; doi:10.18632/oncotarget.18095)
Supplement: Supplementary file 1 [file oncotarget-08-61318-s001.pdf]

# A functional variant in GREM1 confers risk for colorectal cancer by disrupting a hsa-miR-185-3p binding site

## SUPPLEMENTARY MATERIALS

**Supplementary Table 1: Basic information of the candidate SNPs selected for genotyping**

| Position | SNP ID | Gene | Region | MAF * |
|---|---|---|---|---|
| Chr15: 33023486 | rs12915554 | GREM1 | 3' UTR | 1.04% |
| Chr15: 33025507 | rs146588909 | GREM1 | 3' UTR | 1.56% |
| Chr15: 33024560 | rs117841568 | GREM1 | 3' UTR | 2.08% |

*: MAF calculated from targeted sequencing data.
